# Comparative Characterization of Human Antibody Response Induced by BNT162b2 Vaccination vs. SARS-CoV-2 Wild-Type Infection

**DOI:** 10.3390/vaccines10081210

**Published:** 2022-07-29

**Authors:** Theano Lagousi, John Routsias, Maria Mavrouli, Ioanna Papadatou, Maria Geropeppa, Vana Spoulou

**Affiliations:** 1 Immunology and Vaccinology Research Laboratory and Infectious Diseases Department “MAKKA”, First Department of Paediatrics, “Aghia Sophia” Children’s Hospital, Athens Medical School, 11527 Athens, Greece; iopapadatou@med.uoa.gr (I.P.); mgeropeppa@med.uoa.gr (M.G.); vspoulou@med.uoa.gr (V.S.); 2 Athens Medical School, University Research Institute of Maternal and Child Health and Precision Medicine, National and Kapodistrian University of Athens, 11527 Athens, Greece; 3 Department of Microbiology, Athens Medical School, 11527 Athens, Greece; jroutsias@med.uoa.gr (J.R.); mmavrouli@med.uoa.gr (M.M.)

**Keywords:** SARS-CoV-2, COVID-19, BNT162b2, humoral immune response, antibodies

## Abstract

Humoral immunity after SARS-CoV-2 immunization or natural infection is thought to be evanescent. In our study, we aimed to longitudinally characterize the kinetics of antibody titers after dual BNT162b2 immunization or wild-type infection. Vaccinated and recovered individuals displayed distinct antibody kinetics, as convalescents had detectable RBD-, S1-specific, and neutralizing IgG antibody titers two weeks post-infection that gradually increased longitudinally, while RBD-, S1-specific, and neutralizing IgG were detected in vaccinees after the first dose, increased significantly 3 weeks post the second dose and decreased significantly 4–5 months thereafter. Neutralizing IgG was significantly higher initially in convalescent individuals; however, vaccines displayed significantly higher neutralizing antibodies 4–5 months post the second dose. In both groups, there was a strong negative association between elapsed time and antibody levels. The avidity of anti-RBD antibody titers increased significantly in patients longitudinally, while in vaccinees initially increased, with subsequent decrease, remaining however higher than antibody avidity of recovered individuals at all time-points. Anti-RBD antibodies were strongly correlated with neutralizing and anti-S1 antibodies in both groups at all time-points. This study facilitates our further understanding of immune response to SARS-CoV-2 and vaccines.

## 1. Introduction

BNT162b2 (Pfizer/BioNTech), a lipid-nanoparticle-formulated mRNA vaccine encoding the SARS-CoV-2 full-length Spike-protein, was the first vaccine that received emergency use authorization by both FDA and EMA against COVID-19 pandemic [1]. Despite its high efficacy in immunocompetent individuals [2], the duration of BNT162b2 protection remains to be defined. Vaccine efficacy has been correlated with the level of neutralizing antibodies, and levels of anti-S and anti-RBD antibodies, although to a lesser extent [3,4]; however, the threshold of protection is not yet clear.

Following natural infection, anti-SARS-CoV-2 antibodies wane over time, similarly to other human coronaviruses (seasonal, SARS-CoV-1, MERS) [5]. There are reports showing that wild-type infection may prevent secondary infection for at least 7 months, signifying a reference point for comparison with vaccine efficacy and immunogenicity [6].

Considering continuous COVID-19 high global incidence and the emergence of more transmissible SARS-CoV-2 variants, data on longer-term vaccine efficacy and immunity following wild-type infection are essential for clarifying the level of herd immunity and the necessity of further booster doses. In our study, we aimed to assess and characterize the humoral immune response to vaccination at different time-points and compare it to immune response following wild-type infection at roughly matched time-points.

## 2. Materials and Methods

### 2.1. Study Population

The study was performed between March 2021 and December 2021. SARS-CoV-2 infection was confirmed by real-time RT-PCR performed with negative and positive internal controls, according to the World Health Organization guidelines. Previous COVID-19 infection was excluded using Elecsys^®^ Anti-SARS-CoV-2 (Roche Diagnostics, Rotkreuz, Switzerland) reagent on a Cobas e 411 immunoassay analyzer for the semiquantitative detection of total antibodies (IgA, IgM, and IgG) against SARS-CoV-2 nucleocapsid protein according to the manufacturer’s instructions, before the administration of the first dose of the vaccine. Participants were selected with comparable demographic characteristics (as shown in Table 1). Subjects with comorbidities were excluded. Sera were collected on D21 (1–2 days before the second BNT162b2 dose) (n = 36), D50 (~30 days after the second BNT162b2 dose) (n = 32) and D140–170 (~120–150 days after the second BNT162b2 dose) (n = 26) for SARS-CoV-2 naïve vaccinated individuals. For SARS-CoV-2 recovered patients, serum samples were obtained on D15–30, median 21.5 (n = 38), D31–60, median 39 (n = 38) and D120–160, median 149, (n = 34) post disease onset. Blood samples were obtained at the time-points stated above from consecutive individuals. All subjects provided informed consent before entering the study. The study protocol has been approved by the Ethical Committee of Aghia Sophia Children’s Hospital.

### 2.2. RBD- and S1-Specific Antibody Titers

Nighty-six-well plates (Nunc Maxisorp, Rochester, NY, USA) were coated with receptor-binding domain (RBD) (334–527 aa) (purity > 95% by SDS-PAGE) (Abclonal, Wobrun, MA, USA), and Spike protein S1 (13–685 aa) (purity > 90% by SDS-PAGE) (Abclonal) at a concentration 2.5 μg/mL and 1.5 μg/mL, respectively, suspended in Phosphate Buffered Saline (PBS). After blocking with PBS containing 2% Bovine Serum Albumin (BSA) at 37 °C for 30 min, diluted serum samples (1/100 in 2% BSA PBS) were added and incubated for 1 h at 37 °C. Each serum sample was tested against BSA (0.01% PBS) to exclude non-specific binding. Detection of specific human antibodies (IgG) was performed by using alkaline phosphatase-conjugated goat anti-human IgG (Jackson ImmunoResearch Laboratories, West Grove, PA, USA, 1/3000) antibody diluted in PBS/BSA. Optimal incubation duration was determined with preliminary experiments, while chessboard titration tests were used for optimizing the concentration of the coated antigens and plasma dilutions for this specific ELISA. Antibody-binding was assessed with the substrate 4-nitrophenyl-phosphate-disodium salt hexahydrate (Sigma Chemicals, Saint Louis, MI, USA) at 405 nm (Chromate reader, Awareness Technology, Palm City, FL, USA). The cut-off value was determined as the mean plus 2 standard deviations (SD) of a pool of general population age-matched controls. Results were presented using GraphPad Prism (version 7.0, GraphPad Software, San Diego, CA, USA).

### 2.3. Avidity Assay of Anti-Peptide Antibodies

An enzyme immunoassay for evaluating avidity based on the urea dissociation of low-avidity antigen-antibody complexes was performed. ELISA plates coated with different protein antigens were incubated with all collected sera at a dilution of 1:100. The avidity of antigen-specific antibody in serum samples was measured using the same ELISA protocol as the one described above, except for a modification in the first washing step following incubation with the primary antibody. These three washes were performed using a washing buffer of 200 μL of 6 M urea diluted in PBS and the plates were allowed to soak during each washing step for at least 4 min [7]. All samples were tested twice, by both the above-mentioned ELISA and the modified one. Avidity Index (AI%) was expressed as follows: AI%  =  (OD mean value from urea treated sample divided by the OD mean value from the non-treated) multiplied by 100.

### 2.4. Neutralization Assay

Neutralizing antibodies (NAbs) against SARS-CoV-2 were measured using the Abclonal SARS-CoV-2 Neutralizing Antibody Screening Kit (RBD) (RK04149), following manufacturers’ instructions. This kit utilizes a surrogate virus neutralization assay for the indirect detection of potential SARS-CoV-2 NAbs in the blood, based on assessing the antibody (independent of class)-mediated inhibition of SARS-CoV-2 S-RBD protein binding to its human host receptor ACE2.

### 2.5. Statistics

Statistical analysis was performed using GraphPad Prism 6. Antibody titer analysis was performed on log-transformed data. Normality was checked with D’Agostino-Pearson and Kolmogorov-Smirnov tests. Unpaired *t*-test was used for comparisons between different groups. Statistical significance was set at a *p*-value of <0.05.

## 3. Results

During the study period, serum samples were obtained from 36 SARS-CoV-2 naïve immunized individuals and 38 SARS-CoV-2 recovered individuals at various timepoints following immunization or infection, respectively. The participants’ demographic characteristics are described in Table 1.

### 3.1. Κinetics of Anti-RBD, Anti-S1, and Neutralizing Antibodies in Convalescent and Vaccinated Individuals

**Figure 1:**
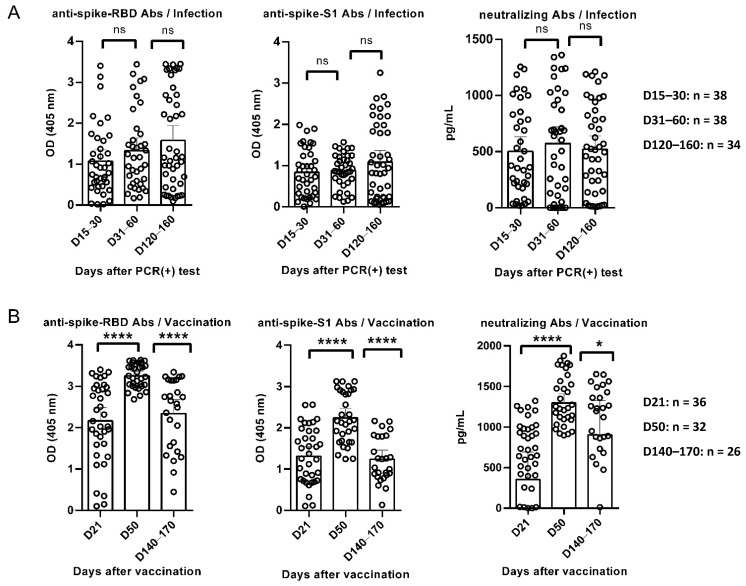
Kinetics of anti-RBD, anti-S1 and neutralizing antibodies (NAbs) in: (**A**) SARS-CoV-2 recovered (D15–30: n = 38, D31–60: n = 38, D120–160: n = 34) and (**B**) vaccinated individuals (D21: n = 36, D50: n = 32, D140–170: n = 26). Diluted sera (1/100 in 2% BSA PBS) were tested against RBD (2.5 μg/mL) and Spike-protein S1 (1.5 μg/mL). The cut-off value was determined as the mean optical density plus 2 standard deviations (SD) of a pool of general population age-matched controls. Each symbol represents the optical density of a serum sample at 405 nm. NAbs against SARS-CoV-2 were measured using the Abclonal SARS-CoV-2 Neutralizing Antibody Screening Kit (RBD) (RK04149), following manufacturers’ instructions. NAbs are measured in pg/mL. * *p* < 0.05, **** *p* < 0.0001, ns: not significant.

Distinct antibody kinetics were recorded between SARS-CoV-2 recovered and vaccinees. Anti-RBD antibodies were detected on D15–30 in convalescent subjects and gradually increased between different time-points, although without statistical significance. Similar trends were found for NAbs and anti-S1 antibodies, which remained almost stable until D120–160 (Figure 1A). High levels of anti-RBD antibodies were detected on D21 for vaccinated individuals; they significantly increased on D50, while found significantly decreased on D140–170. Similar kinetics were found for anti-S1 antibodies and NAbs at the same time-points (Figure 1B).

To compare humoral immune responses after SARS-CoV-2 vaccination versus infection, we used titers on D21 for vaccinated individuals versus titers on D15–30 for SARS-CoV-2 recovered patients (time-point T1). Similarly, titers on D50 and D140–170 among vaccinated were compared to titers D31–60 and D120–160 among SARS-CoV-2 infected individuals (time-points T2 and T3, respectively). Anti-RBD antibodies among vaccine recipients were significantly higher compared to recovered participants (*p* < 0.0001), even at their lowest recorded time-points, although at T3 this difference was not statistically significant (*p* = 0.058). Anti-S1 antibodies were significantly higher in vaccinated individuals as compared to anti-S1 antibodies for convalescent subjects on T1 and T2 (*p* = 0.0026 and *p* < 0.0001, respectively), while on T3 this difference was not statistically significant (*p* = 0.4615). Levels of NAbs among infected individuals were significantly higher at T1 compared to vaccinees (*p* = 0.0424), although this trend changed longitudinally with NAbs titers among vaccinees being significantly higher at T3 compared to infected individuals (*p* < 0.0001).

**Figure 2:**
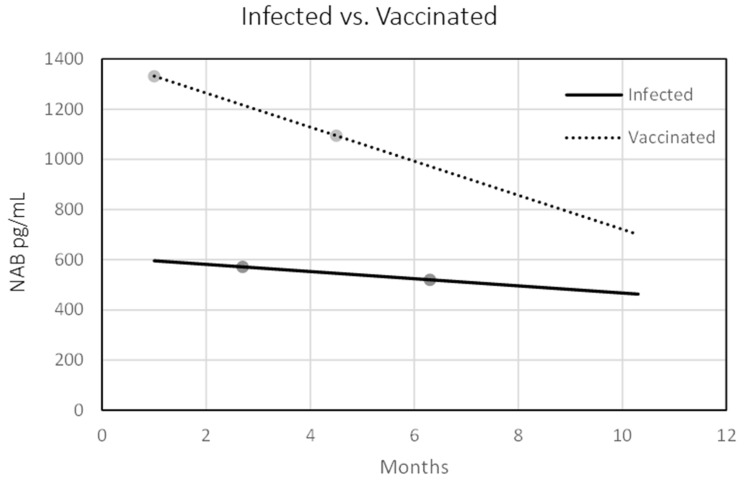
Scatter plot of elapsed time since second BNT162b2 vaccine dose (dotted line) and infection (continuous line) on the x-axis and NAbs titers on the y axis.

We then used linear regression models to assess the correlation between elapsed time and levels of NAbs in both vaccinated and convalescent individuals. In the vaccinees, higher initial antibody titers were recorded, which quickly dropped, decreasing by 5–6% each passing month. Conversely, in the previously infected population, initial titers were lower (intercept of 357 at time zero), but decreased at a much slower rate, by ~2.5% every month. In both groups, there was a strong association (*p* < 0.001) between elapsed time and antibody titers: the decay factor was −67.9 in vaccinees, while for convalescent participants the decrease factor was −14.3 (Figure 2).

### 3.2. Kinetics of Anti-RBD Antibody Avidity

**Figure 3:**
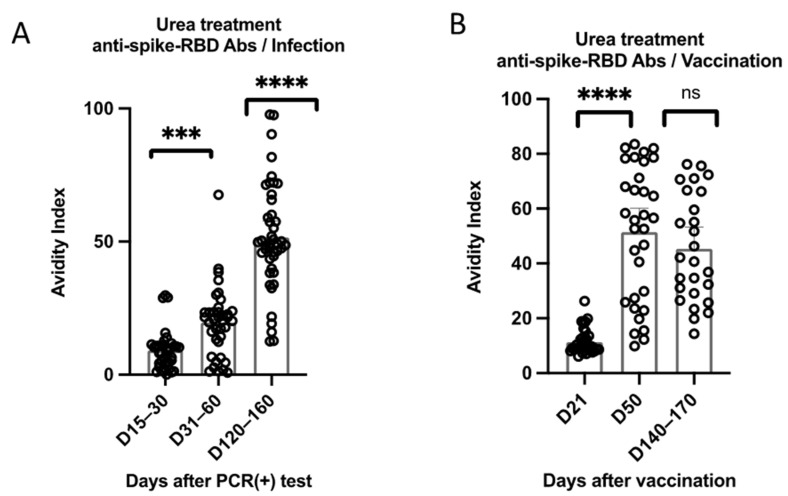
Kinetics of anti-RBD antibody avidity in (**A**) SARS-CoV-2 convalescent and (**B**) immunized individuals. Avidity measurements were based on the urea dissociation of low-avidity antigen-antibody complexes, using the previously described ELISA protocol, with the modification of washing steps by using 200 μL of 6 M urea diluted in PBS. Avidity Index (AI%) was expressed as follows: AI%  =  (OD mean value from urea treated sample divided by the OD mean value from the non-treated) multiplied by 100. *** *p* < 0.001, **** *p* < 0.0001, ns: not significant.

The avidity of anti-RBD antibodies was evaluated using urea dissociation assays. The avidity of anti-RBD antibodies significantly increased between different time-points among recovered individuals. Notably, on D15–30, the avidity was relatively low; it significantly increased on D31–60, and continued to significantly increase on D120–160, despite the more gradual non-significant increase of the anti-RBD antibodies at the corresponding time-points (Figure 3).

On the other hand, the avidity of anti-RBD antibodies significantly increased on D50 among vaccinated individuals but was found to decrease on D140–170, although not less significantly (*p* = 0.008).

Comparing the avidity of anti-RBD between the two groups at different time-points, the avidity of anti-RBD antibodies among vaccinees was significantly higher compared to infected individuals at T1 and T2 (*p* < 0.0001), although at T3 this difference was less significant (*p* = 0.049).

### 3.3. Correlations of Anti-SARS-CoV-2 Antibody Titers

**Figure 4:**
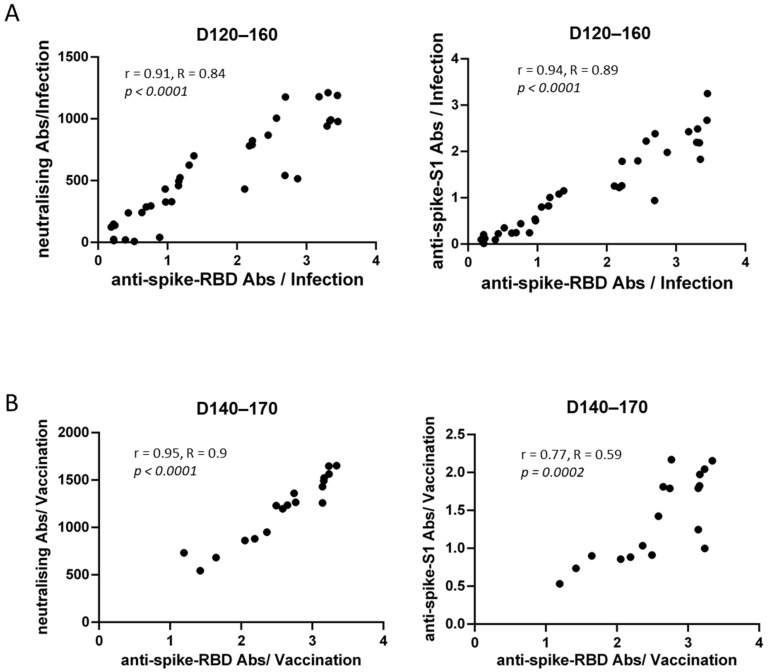
Correlation of anti-RBD antibody titers with NAbs and anti-S1 antibody titers in (**A**) SARS-CoV-2 recovered and (**B**) vaccinated individuals, on D120–160 and D140–170, respectively. Each dot represents a specific serum sample.

The correlation between SARS-CoV-2 neutralizing antibody and anti-RBD antibody titers was assessed. We found a strong correlation between these two antibody titers in both groups of our study at all time-points (r > 0.7). Figure 4 shows the corresponding correlations for both groups at T3; (r = 0.91, R = 0.84, 95% CI 0.84–0.95, *p* < 0.0001) for the convalescent sera on D120–160 and r = 0.94, R = 0.89, 95% CI 0.87–0.97, *p* < 0.0001 for the vaccinees on D140–170 (Figure 4).

Following, a strong correlation between antibody titers against RBD and S1 protein was found (r > 0.7) at all time-points. This correlation was higher among the convalescent sera compared to the vaccines at T3 (r = 0.95 and r = 0.77, respectively) (Figure 4).

## 4. Discussion

The humoral immune response among individuals following either vaccination or documented COVID-19 was assessed in our study at three different time-points after immunization or wild-type infection. Among SARS-CoV-2 naïve individuals who received the Pfizer-BioNTech mRNA vaccine, high initial anti-RBD antibody levels were recorded, followed by a significant increase after the second dose and a significant decrease thereafter. Lower initial anti-RBD antibody levels were recorded among patients who had been infected with SARS-CoV-2, which continued to increase, although not statistically significantly, during the study period.

Similar trends among vaccine recipients and infected individuals were observed for anti-S1 antibodies. Importantly, a strong correlation between anti-RBD and anti-S1 antibodies was recorded between the two groups at all different time-points. Anti-RBD antibodies, that indirectly represent NAbs, were strongly correlated with actual NAbs, suggesting that anti-RBD antibodies bind to the ACE receptor, thus leading to viral neutralization similarly to NAbs. This hypothesis is further supported by previous studies, as Li et al. have reported a strong correlation between anti-RBD antibodies with NAbs [8]. Furthermore, it has been demonstrated that neutralizing antibody titers could serve as a predictive biomarker of protection from symptomatic SARS-CoV-2 infection [3]. Therefore, measuring anti-RBD antibodies may be an easy and reliable tool to assess serological immunity at a population level.

Our findings are in accordance with previous reports showing that humoral response following BNT162b2 vaccination is characterized by a steeper increasing slope of antibodies against both RBD and S1 domain after the second dose in vaccinated compared to infected individuals, followed by a steeper decreasing trend thereafter [9]. Notably, a persistent but substantial waning of antibody responses at 6 months following the second immunization with the BNT162b2 vaccine has been previously reported, especially among men, the elderly, and immunocompromised individuals, while a significant proportion of vaccinees have antibody titers below the detection limit [10,11,12,13]. NAbs significantly increased one month after the second dose among vaccinees, but significantly decreased 4–5 months following the second dose. Among recovered individuals, NAbs were detected 2–4 weeks post-infection, increased during 1–2 months later, while slightly decreased thereafter although in a more constant way. This finding agrees with other studies showing a faster decay of NAbs among vaccinated individuals than in those who were previously infected [14]. Higher levels of all immune markers were correlated with a reduced risk of symptomatic infection, although a definitive individual threshold of protection does not exist.

SARS-CoV-2 antibody titers decline more slowly 4 to 6 months after infection [15]. Antibodies against S-protein following wild-type infection were relatively stable over 6 months [6,16,17]. Previous studies demonstrated that antibody titers may be durable for up to 14 months after wild-type infection and reported that these humoral responses did not originate from plasmablasts, but from antigen-specific memory B cells [8,18,19]. A previous study has shown that immune memory remained measurable in the majority of subjects for more than 8 months after infection. This finding suggests that most convalescents may develop long-lasting immunity against secondary COVID-19, although the emergence of new variants raises several concerns, albeit severe disease remains unlikely [6].

Rapidly decreasing anti-SARS-CoV-2 antibody levels following vaccination indicate that humoral immunity might be transient, implying the necessity of a booster dose [20]. In contrast, SARS-CoV-2 proteins and nucleic acids may be detected in the intestine for at least two months, augmenting the ongoing antibody production in germinal centers [21]. This may explain the slight, but non-significant, increase in anti-SARS-CoV-2 antibodies over a more than 5-month period post-infection observed in our study.

Avidity significantly increased over time between different time-points among infected individuals, suggesting a maturation of immune response over time [22]. Antibody avidity increased following the second dose, albeit decreased on D140–170, although not significantly.

NAbs titers serve as a predictive marker of immune protection and provide an evidence-based model of immune protection against SARS-CoV-2 that will facilitate the development of efficient vaccination policies for harnessing the future trajectory of the pandemic [23]. Modeling of the decay of the neutralization titers over an almost 5-month period following vaccination suggests that most vaccinated individuals will eventually become vulnerable to SARS-CoV-2 infection, although protection from severe COVID-19 may be largely preserved [3].

The severity of SARS-CoV-2 infection is thought to influence the intensity and duration of the humoral immune response, as severe disease has been linked with higher antibody titers early during convalescence, compared to milder or asymptomatic disease [24,25]; however, these differences tend to diminish longitudinally and no differences in SARS-CoV-2-specific and neutralizing antibody titers are detected between different severity groups after 3–6 months [25,26,27]. In our study cohort, PCR-positive for SARS-CoV-2 infection individuals were asymptomatic or presented with mild symptoms, while none of them required hospitalization. Therefore, disease severity could not have been a possible co-factor of humoral response in our cohort.

Our study has several limitations. Firstly, the study included a small number of participants; however, the participants were followed up for several months. Therefore, our results demonstrate humoral response on a longitudinal basis. Furthermore, the study was carried out before the implementation of the booster dose in the vaccination schedule. However, we assessed the humoral immune response following a two-dose vaccine schedule and found that it rapidly decreased longitudinally compared to wild-type infection. Such findings suggest that previously uninfected individuals, vaccinated with a primary two-dose schedule may benefit from an additional booster dose, further supporting the current immunization programs in many countries including booster doses. Besides, exclusion criteria involved major comorbidities (malignancies, immunosuppression, chronic kidney disease, liver failure, genetic syndromes), as the purpose of our study was to investigate the kinetics of immune response in a cohort of healthy individuals; humoral immune response among high-risk individuals was beyond the scope of this study. Additionally, antibody titers were not measured using a commercially available ELISA kit. Nevertheless, an in-house ELISA was performed, with comparable specificity and sensitivity to currently available ones [28]. Besides, blood samples, especially among patients, were obtained during a quite wide time period, however, sera were divided into three different sub-groups with roughly matched time-points to allow comparisons between groups.

## 5. Conclusions

Collectively, our study describes the kinetics of humoral response after primary immunization series with two doses of BNT162b2 or wild-type after on a longitudinal basis. Vaccinated and convalescent individuals showed distinct antibody kinetics, as vaccinees achieved higher antibody titers shortly after immunization, but waned substantially in the following months, while recovered individuals displayed a gradual increase of antibody titers longitudinally. On the contrary, vaccines developed robust NAbs 4–5 months after immunization. Elapsed time was associated with the reduction of antibody titers in both groups, while anti-RBD antibodies were correlated with NAbs and anti-S1 antibodies at all time-points.

The clinical implications of decreasing humoral responses after immunization remain unclear and it is necessary to establish antibody titers thresholds that correspond with protection against severe disease. In uninfected healthcare workers completing the two-dose vaccine schedule, a booster dose is a reasonable option to counteract the substantial antibody decline [14]. Considering the ongoing SARS-CoV-2 pandemic and the emergence of new variants as well as the current advice in support of booster vaccinations in several European countries, our data contribute to further understanding the immune response and level of protection following vaccination and infection.

## Figures and Tables

**Table 1:** The demographic characteristics of vaccinated and recovered individuals.

		Vaccinated	Convalescent
n		36	38
Samples included at each timepoint	D21/D15–30	36	38
	D50/D31–60	32	38
	D140–170/D120–160	26	34
Age	Median (in years)	37	39
	Range (in years)	24–52	22–53
Gender	Female (n, %)	21 (58.3%)	22 (57.9%)
	Male (n, %)	15 (41.7%)	16 (42.1%)
Body Mass Index (BMI)	Mean (SD)	25.1 (4.4)	25.7 (4.8)

## Data Availability

Further information about data supporting the reported results, should be directed and will be fulfilled by the corresponding author, upon reasonable request.
